# DISC1-binding proteins in neural development, signalling and schizophrenia

**DOI:** 10.1016/j.neuropharm.2010.12.027

**Published:** 2012-03

**Authors:** Nicholas J. Bradshaw, David J. Porteous

**Affiliations:** Medical Genetics Section, Molecular Medicine Centre, Institute of Genetics & Molecular Medicine, University of Edinburgh, Western General Hospital, Crewe Road South, Edinburgh, Midlothian EH4 2XU, UK

**Keywords:** DISC1, Schizophrenia, Neurodevelopment, Signalling, Synapse, Association studies, APP, Amyloid precursor protein, ATF4, Activating transcription factor 4, BACE1, β-site APP-cleaving enzyme-1, BBS4, Bardet–Biedl syndrome 4, CEP290, Centrosomal protein 290 kDa, CNV, Copy number variation, CRE, cAMP response element, DBZ, DISC1-binding zinc finger, DISC1, Disrupted in schizophrenia 1, Dixdc1, Dishevelled-axin domain containing-1, FEZ1, Fasciculation and elongation protein zeta 1, GluR, Glutamate receptor, GSK3β, Glycogen synthase kinase 3β, Kal7, Kalirin-7, LEF/TCF, Lymphoid enhancer factor/T cell factor, LIS1, Lissencephaly 1, mTOR, Mammalian target of rapamycin, NDE1, Nuclear distribution factor E homologue 1 or Nuclear distribution element 1, NDEL1, NDE-like 1, NRG, Neuregulin, PACAP, Pituitary adenylate cyclase-activating polypeptide, PCM1, Pericentriolar material 1, PCNT, Pericentrin, PDE4, Phosphodiesterase 4, PI3 K, Phosphatidylinositiol 3-kinase, PSD, Post-synaptic density, Rac1, Ras-related C3 botulinum toxin substrate 1, TNIK, Traf2 and Nck interacting kinase

## Abstract

In the decade since *Disrupted in Schizophrenia 1* (*DISC1*) was first identified it has become one of the most convincing risk genes for major mental illness. As a multi-functional scaffold protein, DISC1 has multiple identified protein interaction partners that highlight pathologically relevant molecular pathways with potential for pharmaceutical intervention. Amongst these are proteins involved in neuronal migration (e.g. APP, Dixdc1, LIS1, NDE1, NDEL1), neural progenitor proliferation (GSK3β), neurosignalling (Girdin, GSK3β, PDE4) and synaptic function (Kal7, TNIK). Furthermore, emerging evidence of genetic association (*NDEL1*, *PCM1*, *PDE4B*) and copy number variation (*NDE1*) implicate several DISC1-binding partners as risk factors for schizophrenia in their own right. Thus, a picture begins to emerge of DISC1 as a key hub for multiple critical developmental pathways within the brain, disruption of which can lead to a variety of psychiatric illness phenotypes.

This article is part of a Special Issue entitled ‘Schizophrenia’.

## Highlights

► We review genetic data associating *DISC1* with psychiatric illness. ► DISC1 binding partners include proteins involved in neuronal migration. ► Others are involved in neuronal signalling or synaptic function. ► These binding partners suggest putative disease-related molecular pathways. ► Several are now also implicated in psychiatric illness in their own right.

## 1 Introduction

A key objective of genetics and genomics research into psychiatric illness is to identify perturbed biological pathways and, as a consequence, potential targets for pharmacological intervention. The genetic entrée point need not itself explain a large fraction of the liability to schizophrenia – it is sufficient that genetic abrogation *can* cause schizophrenia. DISC1 and the extended DISC1 pathway illustrate this contention par excellence. DISC1 was identified through a unique family in which a chromosomal translocation event co-segregates strongly with major mental illness (Blackwood et al., 2001; St Clair et al., 1990). This translocation event directly disrupts both a protein coding gene, *DISC1,* and an antisense RNA only gene, *DISC2* (Millar et al., 2000). In the intervening period, the *DISC* locus has been repeatedly implicated in psychiatric illness by genetic linkage, association and mutation detection (reviewed in Chubb et al., 2008, see Table 1 for references and summaries of recent studies). Some studies have also pointed to epistatic interactions between the *DISC* locus and other candidate genes (Burdick et al., 2008; Hennah et al., 2007; Nicodemus et al., 2010). Despite these many confirmatory studies, there are also negative studies (Chubb et al., 2008 and references in Table 1) and, as yet, no firm basis on which to estimate the proportion of genetic liability attributable to the *DISC* locus. The *DISC* locus has appeared as a gene-wide, but not a genome-wide finding in some (Sullivan et al., 2008) but not other (Sanders et al., 2008) studies. The critical issue is what we can learn from the identification of DISC1 regarding the specifics and generalities of the biological underpinning of schizophrenia and other major mental illness.

The DISC1 protein has no known enzymatic activity; rather it exerts its effect on multiple proteins through interaction to modulate their functional states and biological activities in time and space. Many putative interacting proteins have been identified through extensive yeast-2-hybrid screening (Brandon et al., 2004; Camargo et al., 2007; Millar et al., 2003; Morris et al., 2003; Ozeki et al., 2003) and, where these have been examined, a large proportion have been validated by downstream experimentation (reviewed in Chubb et al., 2008). These multiple interactions, combined with the widespread subcellular distribution of DISC1 (reviewed in Chubb et al., 2008), a complex pattern of protein isoforms (James et al., 2004) and splice variants (Nakata et al., 2009), have led to the suggestion that DISC1 acts as a protein scaffold within the cell, dynamically interacting with and affecting the function of different proteins at different locations and developmental times (Brandon et al., 2009; Porteous, 2008; Porteous and Millar, 2009). *DISC1*-related psychiatric illness is therefore likely to arise through the simultaneous dysregulation of not just one, but more likely several, protein interactions, physiological states and activities, with a consequential complexity and pervasiveness of effect. Identifying the key DISC1 interactors is therefore of exceptional importance in coming to understand the nature of the devastating condition that is schizophrenia, and will facilitate the search for downstream elements which may be susceptible to pharmaceutical intervention.

In this review, we will focus on what is known about the biological functions of DISC1-interacting proteins, with particular attention to aspects of their biology which potentially relate to psychiatric illness, through effects on neurodevelopment, neurotransmission or neurosignalling, along with the emerging genetic evidence implicating many of these as schizophrenia-risk factors in their own right. Whereas a neurodevelopmental component to schizophrenia is well established, and pre-morbid features are recognised, it is typically not until adolescence or early adulthood that the debilitating symptoms emerge. That DISC1 and its pathway of interacting proteins affect both neurodevelopmental pathways and also signalling pathways in the adult brain, suggest that the study of DISC1 genetics and biology may help towards a more unified understanding of schizophrenia and with it the potential to develop rational interventions in the symptomatic adult and or even earlier.

## 2 Roles of DISC1-interacting proteins in neural development

### 2.1 DISC1-binding partners in cytoskeletal functions and neurite outgrowth

**Fig. 1:**
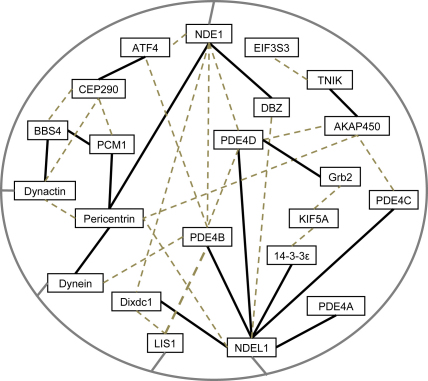
22 known DISC1-interacting proteins, the majority of which are found in or around the centrosome. Proteins which are known to bind directly to each other are linked by thick black lines, while proteins which are known to co-exist in the same complex, but for which a direct-interaction has, to our knowledge, yet to be demonstrated are linked by grey dashed lines. The circle linking dynein, dynactin, LIS1, NDE1 and NDEL1 signifies that these five proteins complex with each other. Note that the DISC1-CEP290, DISC1-AKAP450 and TNIK-AKAP450 interactions have only been shown by yeast-2-hybrid screening and remain to be confirmed, while Grb2 binds only to a single isoform of PDE4D. Data on interactions with DISC1 and between DISC1-binding partners were taken from the following papers: (Beard et al., 1999; Bradshaw et al., 2008, 2009; Brandon et al., 2004; Burdick et al., 2008; Camargo et al., 2007; Chang et al., 2006; Collins et al., 2008; Ewing et al., 2007; Faulkner et al., 2000; Feng et al., 2000; Guo et al., 2006; Hattori et al., 2007; Hirohashi et al., 2006; Hutchins et al., 2010; Kim et al., 2004, 2008b; Kitagawa et al., 2000; McCahill et al., 2005; Millar et al., 2003, 2005; Miyoshi et al., 2004; Morris et al., 2003; Murdoch et al., 2007; Niethammer et al., 2000; Ogawa et al., 2005; Ozeki et al., 2003; Purohit et al., 1999; Sasaki et al., 2000; Sawamura et al., 2008; Sayer et al., 2006; Shinoda et al., 2007; Singh et al., 2010; Smith et al., 2000; Stehman et al., 2007; Sweeney et al., 2001; Tai et al., 2002; Takahashi et al., 2002; Taya et al., 2007; Toyo-oka et al., 2005; Wang et al., 2010).

The complex and intricate task of co-ordinating the microtubule network of neurons, which is vital for maintaining correct development, morphology and migration, is performed in large part by the microtubule organising centre, or centrosome (Higginbotham and Gleeson, 2007). Multiple lines of evidence demonstrate that DISC1 is part of a protein complex at the centrosome (Fig. 1 and references in the legend) and is involved in cytoskeletal processes involved in neuronal migration, including nucleokinesis and neurite outgrowth. LIS1, NDE1, and NDEL1 are a trio of such centrosomal DISC1 interactors (Bradshaw et al., 2009; Brandon et al., 2004; Burdick et al., 2008; Millar et al., 2003; Morris et al., 2003; Ozeki et al., 2003) which play pivotal roles in the progression of the cell cycle, dynein-related transport along microtubules and nucleokinesis (reviewed in Chubb et al., 2008; Wynshaw-Boris et al., 2010). The localisation of NDEL1 and LIS1 in axons is known to be dependent on expression of DISC1 (Taya et al., 2007). Knock-down of either NDEL1 or LIS1 using RNAi in culture leads to reduced neurite outgrowth (Kamiya et al., 2006; Shim et al., 2008; Taya et al., 2007), while granule neurons from heterozygous *NDEL1* or *LIS1* knock-out mice show impaired migration in *in vitro* assays (Toyo-oka et al., 2005). NDEL1 is also known to play a role in axon regeneration after injury (Toth et al., 2008) and has an additional DISC1-modulated function as a cysteine endopeptidase (Hayashi et al., 2005) which appears to be important for regulation of neurite outgrowth (Hayashi et al., 2010). Although highly similar in amino-acid sequence to NDEL1, the role of NDE1 in many of these processes has yet to be determined.

DISC1 is also known to be involved in transport along microtubules to the distal parts of axons as part of a ternary complex with kinesin-1 and the adaptor protein Grb2 (Shinoda et al., 2007; Taya et al., 2007). Cargo transported in this manner includes the DISC1 interactors LIS1, NDEL1 and 14-3-3ɛ (Taya et al., 2007). The interaction of DISC1 with Grb2 may also be required for neutrophin-3-related axon elongation (Shinoda et al., 2007). Of potential therapeutic relevance, expression of *GRB2* mRNA is known to be upregulated following electroconvulsive seizure, an established anti-depression therapy (Newton et al., 2004). FEZ1, another DISC1-interacting protein (Miyoshi et al., 2003), is known to be involved in the activation of the kinesin-1 motor protein (Blasius et al., 2007; Fujita et al., 2007), regulation of neurite outgrowth (Fujita et al., 2007) and the establishment of neuronal polarity (Ikuta et al., 2007). DISC1-FEZ1 interaction is enhanced during neurodifferentiation, and expression of the FEZ1-binding domain of DISC1 has a dominant negative effect on neurite outgrowth in a cellular model (Miyoshi et al., 2003), implying co-operation of DISC1 and FEZ1 in this signalling pathway.

At the centrosome, DISC1 also interacts with the scaffold protein pericentrin (also known as kendrin, Miyoshi et al., 2004), a molecule that is known to play important roles in microtubule nucleation and aster formation (reviewed in Delaval and Doxsey, 2010) in a seemingly DISC1-dependent manner (Shimizu et al., 2008). Intriguingly, mutations in the *PCNT* gene, which encodes pericentrin, are heavily implicated in a form of dwarfism associated with reduced brain size, suggesting it to be important for neurodevelopment (Griffith et al., 2008; Rauch et al., 2008). Recruitment of pericentrin to the centrosome is essential for correct microtubule organisation and is facilitated by another large scaffold protein PCM1 (Dammermann and Merdes, 2002). PCM1 in turn is recruited co-operatively by interacting proteins DISC1 and BBS4 (Kamiya et al., 2008). Centrosomal PCM1 is known to be required for correct axon morphology (Calderon de Anda et al., 2010) and embryonic neurogenesis (Ge et al., 2010). Intriguingly, the level of localisation of PCM1 to the centrosome in human glial cells is altered by two common *DISC1* amino-acid substitutions, Ser704Cys and Leu607Phe (Eastwood et al., 2010), one possible mechanism by which these alleles lead to DISC1 dysfunction, as measured by brain imaging (Callicott et al., 2005; Di Giorgio et al., 2008; Hashimoto et al., 2006; Prata et al., 2008; Szeszko et al., 2008; Takahashi et al., 2009) and elevated risk of psychiatric illness (Table 1 and references therein).

Other centrosomal interactors of DISC1 include the DISC1-Binding Zinc finger protein (DBZ, also known as Su48 or ZNF365). DBZ is a brain expressed protein which binds to DISC1, NDE1 and NDEL1 (Camargo et al., 2007; Hattori et al., 2007; Hirohashi et al., 2006; Wang et al., 2006). Co-expression of DISC1 and DBZ results in a reduction in the number of PC12 cells bearing neurites, while expression of the DISC1-binding domain of DBZ lead to reduced neurite outgrowth in mouse primary hippocampal neurons (Hattori et al., 2007).

### 2.2 DISC1-binding proteins in neuronal migration and differentiation within the mouse brain

Results from cell-based models such as those described above beg the question as to how binding partners of DISC1 might be involved in regulating neurodevelopment. Important insights have come from various *in vivo* mouse studies. In the hippocampus, knock-down of DISC1 using shRNA methods have been shown to lead to aberrant positioning and dendritic structure of adult-born neurons (Duan et al., 2007). Intriguingly, the defects caused by one *DISC1* shRNA of mild effect were greatly enhanced by co-expression of an shRNA to knock-down levels of NDEL1 (Duan et al., 2007), strongly implying that these two proteins co-operate together, and consistent with the role of NDEL1 established in cultured cells. Deficiencies in the migration of neurons in the developing cortex can be seen following silencing of *DISC1*, *PCM1* or *BBS4* (Calderon de Anda et al., 2010; Kamiya et al., 2005, 2008) and in *NDE1* and *NDEL1* knock-out mice (Feng and Walsh, 2004; Sasaki et al., 2005) implying that the various DISC1-containing complexes involved in microtubule regulation are critical for cortical development.

Migration defects in cortical neurons can also be caused by silencing the DISC1-interacting protein Dixdc1 or by inhibiting DISC1-Dixdc1 interaction using an interfering peptide (Singh et al., 2010). Intriguingly, Dixdc1 is also an interactor of NDEL1, and a phosphorylation site key to this interaction is required to rescue migration defects caused by suppression of DIXDC1 expression (Singh et al., 2010). Thus, DISC1, Dixdc1 and NDEL1 appear to co-operate in regulating migration in the developing cortex. Another potential member of this pathway is the Alzheimer’s disease-related Amyloid Precursor Protein (APP, Young-Pearse et al., 2010). Knock-down of APP levels by RNAi in the developing cortex leads to migration defects reminiscent of DISC1 knock-down which can be largely reversed by DISC1 over-expression (Young-Pearse et al., 2007, 2010). There is also evidence to suggest that APP is involved in the localisation of DISC1 to the centrosome in the cortex (Young-Pearse et al., 2010).

Both Dixdc1 and DISC1 impact on the *Wnt*-signalling pathway. Silencing of DISC1 or Dixdc1 reduces lymphoid enhancer factor/T cell factor (LEF/TCF) mediated transcription and thus differentiation of neural progenitors (Mao et al., 2009; Singh et al., 2010). These effects caused by down-regulation of DISC1 expression can be rescued by expression of Dixdc1 and *vice versa*. The key linking molecules are Glycogen Synthase Kinase 3β (GSK3β) and β-catenin. The kinase activity of GSK3β is inhibited on binding to DISC1 (Mao et al., 2009), preventing degradation of β-catenin and allowing it to translocate to the nucleus where it stimulates transcription of neurogenesis-related genes. These effects of DISC1/Dixdc1 silencing can be rescued by expression of β-catenin or by inhibiting GSK3β (Mao et al., 2009; Singh et al., 2010). Also, and of clinical relevance, GSK3β is a well-established target for lithium chloride, widely used in the management of bipolar disorder (Ross and Margolis, 2009). GSK3β-specific inhibitors can also rescue hyperlocomotion in open field tests observed in mice expressing the DISC1-L100P mutant or in which DISC1 has been silenced in the hippocampus (Lipina et al., 2010a; Mao et al., 2009) as well as pre-pulse and latent inhibition deficits in the L100P mouse (Lipina et al., 2010a). Intriguingly, GSK3 activity is also regulated by the APP-derived β-amyloid peptide (reviewed in Hernández et al., 2010).

Girdin (also known as KIAA1212, APE, GIV and HkRP1) is another DISC1-interacting protein (Camargo et al., 2007; Enomoto et al., 2009; Kim et al., 2009), over-expression of which leads to adult-born neurons of the dentate gyrus displaying enhanced dendritic growth, increased dendritic number and over-extended migration into the outer granule cell layer and molecular layer (Kim et al., 2009), mirroring the effects of DISC1 depletion (Duan et al., 2007). Incorrect neuronal localisation and impaired mossy fibre development are also seen in *girdin* knock-out mice (Enomoto et al., 2009). These effects of girdin appear to be mediated via its ability to bind to and increase the activity of the serine/threonine kinase Akt (Anai et al., 2005). DISC1 depletion increases Akt activity (Hashimoto et al., 2006) at least in part through binding to girdin and preventing its Akt-stimulating activity (Kim et al., 2009). In agreement with this, use of rapamycin to inhibit mTOR, which lies downstream of Akt signalling, can rescue the neuronal abnormalities caused by Girdin over-expression or DISC1 knock-down (Kim et al., 2009). Akt is also a negative modulator of GSK3β, although an inhibitor of GSK3β was not seen to rescue these girdin-related developmental defects (Kim et al., 2009).

### 2.3 DISC1-binding partners at the post-synaptic density

In addition to modulating the proliferation, migration and integration of neurons, it can also be postulated that proteins of the DISC1 complex might impact upon major mental illness by modulation of synaptic transmission. In support of this, DISC1 and several of its binding partners, including citron, PDE4B, LIS1, NDE1 and NDEL1, have been found to localise to the post-synaptic density (PSD, Bradshaw et al., 2008; Clapcote et al., 2007; Furuyashiki et al., 1999; Kirkpatrick et al., 2006; Niethammer et al., 2000; Zhang et al., 1999). To date however, relatively little is understood of the synaptic functions of these proteins.

In contrast, more is known about the role of the PSD-localised DISC1 interactor TNIK (Camargo et al., 2007), a kinase expressed in the mouse hippocampus (Wang et al., 2010) whose activity is involved in regulation of the cytoskeleton (Fu et al., 1999). DISC1-binding inhibits the kinase activity of TNIK, leading to the degradation of several key PSD proteins, including the important structural protein PSD95, and modulating the surface expression of glutamate receptor 1 (GluR1, Wang et al., 2010). More generally, knock-down of *DISC1* expression leads to an increase in the formation of spines and GluR1-expessing synapses in mature rat neurons, a process dependent on its interaction with kalirin-7 (Kal7, Hayashi-Takagi et al., 2010). Kal7-dependent regulation of spine formation occurs through its activity as a GDP/GTP exchange factor for Rac1 (Xie et al., 2007), and DISC1 appears to inhibit its activity by binding to Kal7 and PSD95 (Hayashi-Takagi et al., 2010). Activation of NMDA receptors causes dissociation of DISC1, Kal7 and PSD95, making Kal7 available to modulate Rac1 and thus spine structure (Hayashi-Takagi et al., 2010). Thus DISC1 appears to modulate the formation of PSD complexes and dendritic spines through regulation of TNIK, Kal7 and Rac1.

Other DISC1 interactors of potential importance at the synapse include APP (Young-Pearce et al., 2010), given its involvement in spine formation (Lee et al., 2009) and enhancement of NMDA receptor activity (Hoe et al., 2009). Another DISC1 interactor, Activating Transcription Factor 4 (ATF4 or CREB2, Millar et al., 2003; Morris et al., 2003; Sawamura et al., 2008) is known to bind to GABA_B_ receptors in the synapse (Nehring et al., 2000; Vernon et al., 2001; White et al., 2000), and its transport from the synapse to the nucleus, where it acts as a transcriptional repressor, is implicated in long-term depression and memory (Lai et al., 2008).

## 3 Roles of DISC1-interacting proteins in signalling

Another important theme in DISC1 biology is its role in a wide variety of signalling pathways, including the GSK3β and Akt pathways discussed above in Section 2.2. A third such pathway involves signalling by the ubiquitous secondary messenger molecule cAMP. The phosphodiesterase 4 family of enzymes degrade cAMP (reviewed in Houslay and Adams, 2003) and isoforms from all four PDE4 subtypes (PDE4A-D) have been demonstrated to interact with DISC1 (Millar et al., 2005; Murdoch et al., 2007). DISC1 binds PDE4 in a low-activity conformation (Millar et al., 2005; Murdoch et al., 2007) and PDE4 activity is diminished in mice with a mutation, Q31L, in a PDE4-binding site on DISC1 (Clapcote et al., 2007). Downstream effects of DISC1–PDE4 interaction remain to be determined, but are likely to include regulation of the activity of cAMP-dependent Protein Kinase A (PKA), substrates of which include the DISC1-interactors NDE1 (Bradshaw et al., 2008) and ATF4 (Elefteriou et al., 2005). In support of this, over-expression of DISC1 exaggerates the repression of CRE-dependent gene transcription caused by ATF4 in response to PKA (Karpinski et al., 1992; Sawamura et al., 2008). PDE4 is the known target for rolipram and other small molecules which have anti-depressant and anti-psychotic activity in rodent models (Kanes et al., 2007; Maxwell et al., 2004; O’Donnell and Zhang, 2004).

Another family of proteins heavily implicated in major mental illness are the neuregulins and the ErbB family of receptors for which cleaved NRG domains act as ligands (reviewed in Schmitt et al., 2008). Intriguingly, NRG1 and NRG2 signalling is seen to increase the expression of a specific DISC1 isoform in a process dependant on the activity of BACE to cleave neuregulins, forming extracellular peptide ligands (Seshadri et al., 2010). This pathway appears to be mediated by PI3 K/Akt signalling and is transcription-dependent (Seshadri et al., 2010). Downstream effects of NRG1 signalling include inducing the expression and activity of ATF4 (Talukder et al., 2000). Expression of DISC1 also appears to be upregulated following signalling by the neuropeptide PACAP, which additionally stimulates interaction of DISC1 with DBZ (Hattori et al., 2007). DISC1 is also implicated in dopamine signalling, which is altered in several DISC1 mouse models (Ayhan et al., 2010; Lipina et al., 2010b; Niwa et al., 2010). Silencing of DISC1 in rat striatal neurons leads to loss of dopamine receptor-expressing cilia (Marley and von Zastrow, 2010).

In summary, it is increasingly apparent that DISC1 is not simply a scaffold for the formation of protein complexes, but more an active hub for regulating divergent signalling pathways, including PDE4/cAMP, Akt/mTOR and GSK3β/β-catenin that are each well known to impact upon neurodevelopmental and/or psychiatric illness. An interesting side point is the apparent convergence of the DISC1 pathway with proteins involved in Alzheimer’s disease. DISC1 is now known to interact with APP (Young-Pearse et al., 2010), from which the β-amyloid peptide is derived, along with the related APLP1 protein (Millar et al., 2003). DISC1 also inhibits the activity of GSK3β (Mao et al., 2009), which is modulated by and may also modulate β-amyloid peptides (Hernández et al., 2010), and DISC1 levels are indirectly regulated by BACE (Seshadri et al., 2010), the APP-cleaving enzyme. By implication, DISC1 plays a critical role in integrating these otherwise independent pathways, elaborating the details of which represents a key future challenge.

## 4 Genetic evidence implicating DISC1 interactors in schizophrenia and related disorders

Several positive genetic association studies have been reported for genes encoding DISC1 interactors (summarised in Table 2), implying that multiple DISC1-related pathways need to be considered as relevant to risk of psychopathology. *ATF4*, *CIT* (encoding citron), *FEZ1*, *NDE1*, *PAFAH1B1* (encoding LIS1), *PCNT* (encoding pericentrin), *PDE4D*, *TNIK* and *YWHAE* (encoding 14-3-3ɛ) are thus all implicated in schizophrenia, although some of these are single studies or report modest associations that await firm replication, with some studies failing to replicate (Table 3 and references therein). Replication of genetic association between one or more SNPs and major mental illness supports *PDE4B*, *NDEL1* and *PCM1* in their own right (Table 2 and references therein).

It is important here also to distinguish between the strict statistical tests for significance that must be applied to gene-wide or genome-wide test of association and the insight which can be gained from specific mutations in individual cases and families (Mitchell and Porteous, 2009; Porteous, 2008). Thus, in much the same way as *DISC1* was discovered at a translocation breakpoint (Blackwood et al., 2001; Millar et al., 2000; St Clair et al., 1990), *PDE4B* was found to be directly disrupted by a t(1:16) translocation in a proband with schizophrenia, who also had an affected cousin (Millar et al., 2005). Similarly, both deletions and duplications at chromosomal locus 16p13.1, containing the *NDE1* gene, are significantly over-represented in schizophrenia patients in Scottish and other European populations, with a similar deletion also seen in an African–American individual with the condition (Ingason et al., 2011; Need et al., 2009). Ultra-rare missense mutations in patients with schizophrenia have been reported for APP (Jones et al., 1992) PCM1 (Kamiya et al., 2008), and indeed DISC1 (Song et al., 2008). These rare mutations point the finger directly at these genes and associated pathways, and further demonstrate their biological relevance.

Evidence has also been reported of transcripts encoding several of these proteins being either up- or down-regulated in brain tissue of individuals with psychiatric illness, compared to that from healthy controls (Table 4 and references therein). Such differences in mRNA expression could be the result of direct mutation in those genes, or indirectly, due to dysregulation of transcription factors or other modulatory proteins. Additionally, certain *DISC1* SNPs are associated with reduced levels of *FEZ1*, *LIS1* and *NDEL1* transcripts in the hippocampus (Lipska et al., 2006).

Additionally, as one would predict biologically, evidence for epistatic interaction is emerging: three-way interaction between specific SNPs in *CIT*, *DISC1* and *NDEL1* has been reported for schizophrenia (Nicodemus et al., 2010); there is strong statistical interplay between the HEP3 haplotype and NDE1 in the Finnish population (Hennah et al., 2007) and haplotypes of *NDE1* and *NDEL1* show association that is dependent on the Ser or Cys variant at position 704 in DISC1 (Burdick et al., 2008).

Although the emphasis of genetic studies to date has been on their potential pathological impact, it is emerging that common variants of DISC1 and its interactors impact on normal variation and intermediate phenotypes, for example memory tasks (Burdick et al., 2005; Cannon et al., 2005; Hennah et al., 2005) and in quantitative measures of personality and mood (Harris et al., 2010). We have recently reported (Hennah and Porteous, 2009) that common cis-acting variants of *DISC1* modulate expression within normal subjects by up to 20%. Moreover, variants in *DISC1*, *PDE4* and *NDE1* impact on the expression of genes involved in the cytoskeleton, neurosignalling and sensory perception, and are significantly enriched for current drug development targets in psychiatry.

In a similar vein, the recent demonstration of at least fifty different DISC1 transcripts including an abundance that are specific to foetal development and some for which expression is altered in the hippocampus of those suffering from schizophrenia or carrying *DISC1* schizophrenia-risk alleles (Nakata et al., 2009), raised a whole new series of questions about how DISC1 expression is regulated, and with what effect on neurodevelopment and signalling.

## 5 Caveats and limitations

Whereas the growing literature on DISC1 and the DISC1 pathway, as summarised here, provides multiple avenues of promising research to pursue that is relevant to neurodevelopment, signalling and psychopathology, there are gaps and limitations. For example, with respect to the DISC1 interactome derived from yeast-two-hybrid analysis, only a minority of putative DISC1 interactors have been formally tested and confirmed by co-immunoprecipitation or co-localisation in native tissue. Although multiple transcripts and protein isoforms of DISC1 have been described, the functional role of the former and the amino-acid sequence of the later remain to be determined. Evidence from the original family from which DISC1 was identified is consistent with a simple haploinsufficiency model, but in the absence of patient tissue other than lymphoblastoid cell lines, it is not possible to rule out a dominant negative effect of hypothetical truncated or fusion DISC1 protein expression during development or in the adult brain. This family is an example of an ultra rare, in this case unique, genetic event revealing a more general genetic contribution though other genetic variants at the locus. A number of amino-acid substitutions in DISC1 been described and regulatory mutations hypothecated from association evidence, but a comprehensive analysis of all possible mutations awaits the results of large scale resequencing studies. Our understanding of the biological consequences of S704C and L607F, the best studied to date, remain partial. Regarding the cell and animal models used to test for biological effects, these do not as yet model known clinical variants, nor, for obvious reasons, do the models allow testing of psychiatric phenotypes, only at best surrogates and proxies. That said, a still modest, but growing body of evidence is making links between mouse models and human studies through comparative brain histology and imaging (reviewed in Johnstone et al., 2010). Thus, despite the remarkable progress, much remains to be done not just *in vitro* or in model systems, but in clinical studies too.

## 6 Summary and conclusions

The complexities of schizophrenia and related psychiatric illness were never likely to yield to single methodologies or models, but a combined genetic and biological approach offers promise. From what might have been viewed as an unlikely start point, the molecular genetic characterisation of a single family with a high loading for psychiatric illness, the insights from the discovery of DISC1 have been manifold and far-reaching, a paradigm for future work. It is now not so much a question of the role of DISC1 *per se*, but much more about the DISC1 pathway in neurodevelopment and signalling, brought to light through the multiplicity of DISC1-interacting proteins. Mechanistic details remain to be filled in and potential therapeutic targets evaluated. But in the decade since DISC1 was cloned, much progress towards these goals has been made by a combination of genetics, biochemistry, neurobiology and animal models. The next decade promises further exciting prospects to enhance our understanding of the DISC1 pathway to the benefit of patients.

## Figures and Tables

**Table 1:** Studies investigating genetic links between the *DISC* locus or the adjacent *TSNAX* locus and major mental illness, an endophenotype thereof or, in one study, chronic fatigue syndrome published since those reviewed by Chubb et al. (2008). SNPs bracketed together indicate haplotypes. SNPs separated by a hyphen (−) indicate significance when alleles are considered together, but not independently.

Study	Sample	Condition/phenotype	SNP, haplotype, marker or variant	Notes
*Positive genetic association studies*				
Palo et al. (2007)	Finnish families	Psychotic disorders	(rs1655285, rs751229)	Males only
			(rs751229, rs3738401)	Males only
			(rs751229, rs3738401, rs1538977)	Males only, principally those without bipolar spectrum disorder
			(rs1655285, rs751229, rs3738401)	Males only, principally those with bipolar spectrum disorder
		Bipolar spectrum disorders	rs1655285	
			(rs1630250, rs1615409)	Principally those without psychotic disorder
			(rs1655285, rs751229)	Females only
			(rs1000731, rs821616)	
			(rs821616, rs1411771)	
			(rs821616, rs1411771, rs980898)	
	Finnish families with bipolar disorder	General intellectual functioning	rs1615409	Significant by two measures
			rs821616	Significant by one measure
			rs980989	Significant by three measures
		Attention/working memory	rs980989	Significant by three measures
		Verbal learning	rs751229	Significant by two measures
			rs1322784	Significant by one measure
			rs1000731	Significant by one measure
			rs980989	Significant by two measures
		Executive functions	rs821616	Significant by one measure
Kilpinen et al. (2008)	Finnish families	Autism	D1S2709	
		Asperger’s syndrome	rs1322784	Males only
			(rs751229, rs3738401)	
			(rs751229, rs3738401, rs1322784)	Males only
Kim et al. (2008a)	Korean	Schizophrenia with poor concentration	rs821616	
Perlis et al. (2008)	American trios	Bipolar disorder	(rs10495308, rs2793091, rs2793085)	
Saetre et al. (2008)	Danish	Schizophrenia	rs3737597	
	Norwegian	Schizophrenia	rs3737597	
	Swedish	Schizophrenia	rs3737597	
Hennah et al. (2009)	Finnish	Bipolar disorder	rs1538979	Males only
	English	Bipolar disorder	rs821577	Females only
	British/Finnish	Schizophrenia	rs821633-rs1538979	Females only.
Rastogi et al. (2009)	Canadian families	Schizophrenia	(rs11122359, rs701158)	
			(rs6675281, rs11122359)	
			(rs701158, rs821597)	
Schumacher et al. (2009)	German	Schizophrenia and early onset schizophrenia	rs1015101	Females only.
			rs999710	Females only.
			rs4333837	Females only.
		Schizophrenia	18x haplotyes	5 in males only, 11 in females only
			rs1538979	Significant in males when stratified on rs821633 allele
Tomppo et al. (2009b)	Finnish	Social anhedonia	rs821577	
			rs11122381	Females only.
			rs821592	Females only.
			rs821633	Significant when stratified on rs1538979 and rs821577 alleles
Fukuda et al. (2010)	Japanese	Chronic fatigue syndrome	rs821616	Females only.
Harris et al. (2010)	Scottish, elderly	Anxiety scores	rs821577	Lower in males, higher in females
			rs821633	Lower in males, higher in females
		Depression scores	rs821577	Females only
			rs821633	Females only
		Emotional stability scores	rs821577	Females only
			rs821633	Females only
		Neuroticism scores	rs821577	Females only
			rs821633	Females only
Lepagnol-Bestel et al. (2010)	French trios	Schizophrenia	rs6675281	
		Negative symptom scores	rs6675281	
	Algerian trios	Schizophrenia	rs821616	
		Negative symptom scores	rs6675281	
Mouaffak et al. (2010)	French	Ultra-resistant schizophrenia	rs3738401	
Nicodemus et al. (2010)	American	Schizophrenia	rs10744743-rs1411771	rs10744743 is an SNP in the *CIT* gene
Okuda et al. (2010)	Japanese	Major depressive disorder	rs766288	Females only
Schosser et al. (2010)	English	Bipolar disorder	rs2492367	
			rs7546310	
			(rs7546310, rs821597)	
			(rs766288, rs2492367)	
			(rs1000731, rs7546310)	
	British	Major depressive disorder	(rs7546310, rs821597)	

*Meta-analysis of association studies*				
Schumacher et al. (2009)	European	Schizophrenia	rs17817356	

*Negative association studies*				
Arai et al. (2007)	Japanese	Bipolar disorder	Negative	
		Major depressive disorder	Negative	
Sanders et al. (2008)	European ancesrtry	Schizophrenia	Negative	
Lim et al. (2009)	Korean	Autism spectrum disorders		
Houlihan et al. (2009)	Scottish, elderly	Cognitive traits	Negative	
Okuda et al. (2010)	Japanese	Bipolar disorder	Negative	

*Ultra-rare variants*				
Song et al. (2008)	Single patients	Schizophrenia	Point mutations: G14A, R37 W, S90L, R418H, T603I	Not in 10,000 + sequenced controls. S90L seen in two patients
Crepel et al. (2010)	Two brothers	Autism	2 Mb duplication including *DISC* locus	Not in 1577 controls
Williams et al. (2009)	Single patient	Autism spectrum disorder	2 Mb deletion including *TSNAX*/*DISC* locus	
Song et al. (2010)	Single patients	Bipolar disorder	Point mutations: S209R R338Q, R418H, T754S	Not in 10,000 + sequenced controls

**Table 2:** Studies which have found positive evidence of association between variants in genes encoding DISC1-interacting proteins and major mental illness. SNPs grouped together in brackets indicate haplotypes. SNPs separated by a hyphen (−) indicate significance when alleles are considered together, but not independently.

Gene	Study	Condition	Sample	SNP, haplotype or marker	Notes
*ATF4*	Qu et al. (2008)	Schizophrenia	Han Chinese	(rs17001266, rs4894)	Males only
*CIT*	Lyons-Warren et al. (2005)	Bipolar disorder	American	rs203368	
				rs435136	
				(rs435136, hCV3259834)	
				(rs203368, rs435136)	
				(rs203368, rs435136, hCV3259834)	
				(rs278109, rs203368)	
				(rs2285595, rs278109, rs203368)	
				(rs2285595, rs278109, rs203368, rs435136)	
	Nicodemus et al. (2010)	Schizophrenia	American	rs10744743	
				rs3847960-rs203332	
				rs3847960-rs440299	
*CIT-DISC1*	Nicodemus et al. (2010)	Schizophrenia	American	rs10744743-rs1411771	
*CIT-NDEL1*	Nicodemus et al. (2010)	Schizophrenia	American	rs10744743-rs4791707	
*FEZ1*	Rastogi et al. (2009)	Schizophrenia	Canadian	(rs2845846, rs2849222)	
*NDE1*	Hennah et al. (2007)	Schizophrenia	Finnish families	(rs4781678, rs2242549, rs881803, rs2075512)	Females only, conditioned on DISC1 HEP3 haplotype
	Burdick et al. (2008)	Schizophrenia	American Caucasian	(rs8061376, rs4781679, rs3784859, rs12934645)	Amongst DISC1 C704 carriers
*NDEL1*	Burdick et al. (2008)	Schizophrenia	American Caucasian	(rs1391768, rs1391766, rs931672, rs35261231)	Not amongst DISC1 C704 carriers
	Tomppo et al. (2009a)	Schizophrenia	Finnish families	rs17806986	
				(rs17806986, rs1391768, rs1391766, rs3817003)	
	Nicodemus et al. (2010)	Schizophrenia	American	rs4791707	
*PAFAH1B1*	Rastogi et al. (2009)	Schizophrenia	Canadian families	(rs8081803, rs12938775)	
	Nicodemus et al. (2010)	Schizophrenia	American	rs12938775	
*PCM1*	Gurling et al. (2006)	Schizophrenia	British & Icelandic families	D8S261	
			Brisith	D8S261	
				(rs445422, 87366_66, rs370429)	
				(rs454755, rs3780103, rs6991775)	
				(rs454755, 87366_66, rs3780103, rs6991775)	
			Scottish	(rs454755, rs3780103, rs6991775)	
			American trios	D8S261	
	Datta et al. (2010)	Schizophrenia	British	rs208747	
				rs445422	
				rs13276297	
				rs370429	
				14 haplotypes	
			Scottish	rs445422	
	Moens et al. (2010)	Schizophrenia	Swedish	rs13276297	
			European	rs445422	Meta-analysis of populations in Datta & Moens studies
				rs208747	
*PCNT*	Anitha et al. (2009)	Schizophrenia	Japanese	rs2249057	
				(rs9981892, rs2249057)	
				(rs9981892, rs2249057, rs2839222)	
	Numata et al. (2009b)	Major depression	Japanese	rs3788265	
				rs2073376	
*PDE4B*	Pickard et al. (2007)	Schizophrenia	Scottish	(rs2503166, rs583018, rs526772)	Females only
	Fatemi et al. (2008)	Schizophrenia	American Caucasian	rs1354064	
				rs4320761	
				rs1040716	
				rs910694	
				rs1321177	
				rs2144719	
				rs783038	
			African American	rs599381	
				rs1040716	
				rs910694	
	Numata et al. (2008a)	Schizophrenia	Japanese	rs2180335	
				rs910694	
				rs472952	
	Numata et al. (2009a)	Major depressive disorder	Japanese	rs472952	Not replicated in second sample
	Rastogi et al. (2009)	Schizophrenia	Canadian	(rs614350, rs2503174)	
				(rs12068439, rs12743648)	
				(rs2503174, rs1577844)	
	Tomppo et al. (2009a)	Schizophrenia	Finnish families	rs7412571	
				(rs10158178, rs7412571, rs5999235, rs2069278)	
				(rs4503327, rs2503222, rs6588186)	
*PDE4D*	Tomppo et al. (2009a)	Schizophrenia	Finnish families	rs1120303	
				(rs13190249, rs1120303, rs921942, rs10805515, rs10514862)	
*TNIK*	Potkin et al. (2009)	Schizophrenia associated with a quantitative trait	American	rs2088885	
				rs7627954	
*YWHAE*	Ikeda et al. (2008)	Schizophrenia	Japanese	rs34041110	
				rs7224258	
				rs3752826	
				rs11655548	
				rs2131431	
				rs1873827	
				rs28365859	

**Table 3:** Studies which failed to find evidence of association of mental illness with any SNP examined of a gene encoding a DISC1-interacting protein.

Gene	Study	Condition	Population
*ATF4*	Kakiuchi et al. (2007)	Bipolar disorder^a^	Japanese
*DBZ*	Anitha et al. (2009)	Schizophrenia	Japanese
		Bipolar disorder	Japanese
*FEZ1*	Yamada et al. (2004)	Bipolar disorder	Japanese
		Schizophrenia^a^	Japanese
	Hodgkinson et al. (2007)	Schizophrenia	American Caucasian
			African American
	Koga et al. (2007)	Schizophrenia	Japanese
	Nicodemus et al. (2010)	Schizophrenia^b^	American
*GRB2*	Ikeda et al. (2008)	Schizophrenia	Japanese
*KIF5A*	Ikeda et al. (2008)	Schizophrenia	Japanese
*NDE1*	Numata et al. (2008b)	Schizophrenia	Japanese
	Nicodemus et al. (2010)	Schizophrenia^b^	American
*NDEL1*	Kähler et al. (2008)	Schizophrenia	Scandanavian
	Ikeda et al. (2008)	Schizophrenia	Japanese
	Rastogi et al. (2009)	Schizophrenia	Canadian
*PAFAH1B1*	Ikeda et al. (2008)	Schizophrenia	Japanese
	Kähler et al. (2008)	Schizophrenia	Scandanavian
*PCNT*	Numata et al. (2010)	Schizophrenia^a^	Japanese
	Anitha et al. (2008)	Bipolar disorder	Japanese
*PDE4B*	Holliday et al. (2009)	Schizophrenia^c^	Tamil Nadu, India
	Rastogi et al. (2009)	Schizophrenia^a^	Canadian
	Kähler et al. (2009)	Schizophrenia^a^	Scandinavian
		Bipolar disorder	Scandinavian
*YWHAE*	Kähler et al. (2008)	Schizophrenia	Scandanavian

a These studies found nominal association with one or more SNPs, but these did not survive correction for multiple testing.

b Nicodemus et al. were principally looking for evidence of genetic epistasis between genes rather than evidence that individual SNPs were associated with schizophrenia.

c Holliday et al. found a risk locus proximal to *PDE4B* in a ethnically homogenous sample, but found no evidence of association to *PDE4B* itself.

**Table 4:** Studies which have found levels of transcripts encoding DISC1-interacting proteins to be significantly altered in RNA from individuals with major mental illness compared to healthy controls.

Gene	Study	Condition	Associated expression profile
*FEZ1*	Lipska et al. (2006)	Schizophrenia	Reduced in the hippocampus and dorsolateral prefrontal cortex
*NDE1*	Fatemi et al. (2010)	Schizophrenia	Increased in cerebellum
		Bipolar disorder	Increased in cerebellum
		Major depression	Increased in cerebellum
*NDEL1*	Lipska et al. (2006)	Schizophrenia	Reduced in the hippocampus
*PAFAH1B1*	Lipska et al. (2006)	Schizophrenia	Reduced in the hippocampus and dorsolateral prefrontal cortex
*PDE4B*	Numata et al. (2009a)	Major depression	Increased in peripheral leukocytes
*TNIK*	Glatt et al. (2005)	Schizophrenia	Increased in dorsolateral prefrontal cortex
	Matigian et al. (2007)	Bipolar disorder	Increased in lymphoblastoids (relative to healthy monozygotic twin)
